# Tartary Buckwheat (*Fagopyrum tataricum*) Ameliorates Lipid Metabolism Disorders and Gut Microbiota Dysbiosis in High-Fat Diet-Fed Mice

**DOI:** 10.3390/foods11193028

**Published:** 2022-09-29

**Authors:** Ang Li, Jin Wang, Yuanyifei Wang, Bowei Zhang, Zhenjia Chen, Junling Zhu, Xiaowen Wang, Shuo Wang

**Affiliations:** 1 College of Food Science and Engineering, Shanxi Agricultural University, Jinzhong 030801, China; 2 Tianjin Key Laboratory of Food Science and Health, School of Medicine, Nankai University, Tianjin 300350, China; 3 Institute of Medicinal Plant, Shanxi Agricultural University, Jinzhong 030801, China

**Keywords:** tartary buckwheat, lipid metabolism disorders, high-fat diet, gut microbiota, mice

## Abstract

Jinqiao II, a newly cultivated variety of tartary buckwheat (*Fagopyrum tataricum*), has been reported to exhibit a higher yield and elevated levels of functional compounds compared to traditional native breeds. We aimed to investigate the potential of Jinqiao II tartary buckwheat to alleviate lipid metabolism disorders by detecting serum biochemistry, pathological symptoms, gene expression profiling, and gut microbial diversity. C57BL/6J mice were provided with either a normal diet; a high-fat diet (HFD); or HFD containing 5%, 10%, and 20% buckwheat for 8 weeks. Our results indicate that Jinqiao II tartary buckwheat attenuated HFD-induced hyperlipidemia, fat accumulation, hepatic damage, endotoxemia, inflammation, abnormal hormonal profiles, and differential lipid-metabolism-related gene expression at mRNA and protein levels in response to the dosages, and high-dose tartary buckwheat exerted optimal outcomes. Gut microbiota sequencing also revealed that the Jinqiao II tartary buckwheat elevated the level of microbial diversity and the abundance of advantageous microbes (*Alistipes* and *Alloprevotella*), lowered the abundance of opportunistic pathogens (*Ruminococcaceae*, *Blautia*, *Ruminiclostridium*, *Bilophila*, and *Oscillibacter*), and altered the intestinal microbiota structure in mice fed with HFD. These findings suggest that Jinqiao II tartary buckwheat might serve as a competitive candidate in the development of functional food to prevent lipid metabolic abnormalities.

## 1. Introduction

Lipid metabolism disorder implies an abnormal serum lipid profile and hepatic lipid distribution, typically manifesting as metabolic diseases, including hypercholesterolemia, hyperlipidemia, and nonalcoholic fatty liver syndrome (NAFLS), which may lead to a higher susceptibility to obesity, atherosclerosis, and diabetes [1]. Accumulating evidence has suggested that the gut microbial ecosystem is highly relevant to host lipid homeostasis and the development of metabolic diseases [2]. A previous study has revealed that obese subjects have a decreased gut microbiota diversity and increased Firmicutes/Bacteroidetes ratios [3]. It is worth noting that diet is widely considered a significant determinant of the microbial community profile [4]. Therefore, developing a dietary strategy that targets intestinal flora is strongly warranted.

Buckwheat has gained increasing popularity over the past several decades as a gluten-free pseudo-grain, and it has been regarded as an attractive raw material to develop a dietary supplement that benefits metabolic health [5]. Currently, twenty-six buckwheat varieties have been identified, of which only two are widely served as food and medicinal plants: common buckwheat (*Fagopyrum esculentum*) and tartary buckwheat (*Fagopyrum tataricum*) [6]. Meanwhile, tartary buckwheat is widely recommended as a nutritious food choice by research experts compared to the broader planted common buckwheat because tartary buckwheat is a rich source of bioactive components, such as rutin and quercetin, in addition to balanced amino acids [7,8]. Jinqiao II, a newly cultivated variety of tartary buckwheat, has recently attracted considerable attention because it has been reported to exhibit a higher yield and elevated levels of functional compounds (e.g., quercetin), as well as amino acids, in comparison with traditional native breeds [9]. With the growing concern about human health, it is highly necessary to advance the development of this novel species and maximize its practical application in healthcare [10]. Moreover, the protective effects, dose-dependent regulation, and valid thresholds of Jinqiao II tartary buckwheat in terms of metabolic health are poorly understudied. The underlying mechanisms that connect lipid metabolism with gut microbiota have also not been evidently discussed. Thus, the present study was conducted to explore the potential mechanisms of Jinqiao II tartary buckwheat at different dose levels in lipid metabolism regulation and to further investigate the detailed mechanism linking lipid metabolic disturbance to the gut microbial profile.

In our study, C57BL/6J mice were provided with either a normal diet; HFD; or a high-fat diet containing 5%, 10%, and 20% buckwheat for 8 weeks. The serum lipid profile, the inflammatory process, and the levels of hormone release in the mice were detected. The histological condition of the liver and adipose tissue was then evaluated. Next, the relative expression levels of lipid-metabolism-related genes were verified at mRNA and protein levels. Gut microbial analyses were also applied to determine the role of gut microbiota in the buckwheat-mediated prevention of lipid metabolism disorder. The present work may provide valuable evidence for the utilization of this functional alternative prepared from novel Jinqiao II tartary buckwheat to prevent lipid metabolism disorder and related metabolic diseases.

## 2. Materials and Methods

### 2.1. Chemicals and Reagents

Jinqiao II tartary buckwheat (National Certification No. 9279-21) was obtained from the Institute of Agricultural Products Processing, Shanxi Academy of Agricultural Sciences (Taiyuan, China). All analytical-grade chemicals were obtained from Sigma (Shanghai, China).

### 2.2. Experimental Animal Design

Six-week-old male C57BL/6J mice, purchased from Vital River Laboratories (Peking, China), were housed in a temperature-controlled room and humidity-controlled facility with a 12 h light/dark cycle. After 1 week of acclimatization, forty mice were randomly divided into 5 groups (*n* = 8): ND group (normal chow diet containing 10% fat, XTCON50J), HFD group (HFD containing 60% fat, XTHF60), LB group (low-dose buckwheat + HFD), MB group (medium-dose buckwheat + HFD), and HB group (high-dose buckwheat + HFD). All the diets were obtained from Xietong Pharmaceutical Bio-engineering Co., Ltd. (Nanjing, China). In the LB, MB, and HB groups, 5%, 10%, and 20% of the high-fat diet were replaced by buckwheat, respectively, and the calorie intake was controlled to be consistent in each group. The detailed components of the diets are provided in Table S1. All mice were allowed to access feed and water freely. At the end of the 8-week trial, fresh fecal and blood samples were separately collected, and the experimental animals were euthanized to obtain liver and adipose tissues for subsequent physiological and pathological analyses after an overnight fast. All experimental procedures conformed to the protocols of the animal resources center of Nankai University (SYXK-2019-0001).

### 2.3. Biochemical Analysis

The levels of total cholesterol (TC), triglyceride (TG), low-density lipoprotein cholesterol (LDL-C), high-density lipoprotein cholesterol (HDL-C), aspartate aminotransferase (AST), and alanine aminotransferase (ALT) in the serum were measured using commercial kits (Jiancheng Bioengineering Institute, Nanjing, China). The circulating levels of lipopolysaccharide (LPS) and inflammatory factors, including interleukin-6 (IL-6), tumor necrosis factor-alpha (TNF-α), and interleukin-10 (IL-10), were detected using corresponding ELISA kits (Suzhou Calvin Biotechnology Ltd., Suzhou, China).

### 2.4. Histological Evaluation

The liver and white adipose tissue fixed in 10% neutral formalin were subjected to hematoxylin–eosin (H&E) staining. The stained sections were then observed and photographed using a microscope (Olympus, Tokyo, Japan).

### 2.5. Hormone Measurement

The serum levels of leptin, adiponectin, glucagon-like peptide 1 (GLP-1), and peptide YY (PYY) were determined using ELISA kits (Suzhou Calvin Biotechnology Ltd., Suzhou, China) based on the corresponding instructions.

### 2.6. Gene Expression Analysis in the Liver

Trizol reagent was used to extract the total RNA from the liver tissue. Reverse transcription reactions were performed using a RevertAid First Strand cDNA Synthesis Kit (Thermo Scientific, Waltham, MA, USA). The final cDNA products were used as templates for qRT-PCR according to Feng et al. with minor revision [11]. The specific oligo nucleotide primer sequences are shown in Table S2. The relative mRNA level for each lipid-metabolism-related gene was normalized to β-actin. The mRNA expression was calculated using the 2^−ΔΔCt^ method and expressed as a fold change in comparison to the ND group.

### 2.7. Western Blot

Protein samples from the liver tissues of the experimental mice were extracted, homogenized, centrifuged, and quantified using a BCA protein assay kit (Solaibao, Beijing, China) according to previously described approaches [12]. After separation on SDS-PAGE gels, the protein samples were transferred to PVDF membranes and incubated overnight with a primary anti-PPARα antibody (1:1000; SAB, NO. 41359), anti-SREBP1c antibody (1:1000; Bioss, NO. bs-1402R), anti-FAS antibody (1:1000, Abcam, NO. ab82419), and anti-LXRα antibody (1:1000, Boster, NO. BA2757-2). Afterward, the membranes were incubated at room temperature for 1h with the appropriate secondary antibodies and visualized using ECL chemiluminescence.

### 2.8. Gut Microbiota Analysis

Microbial total genomic DNA was extracted, followed by a bacterial 16s rDNA amplification of the V3-V4 fragment using primers 338F/806R. Microbial analyses, including principal coordinate analyses (PCoA), line discriminant analysis (LDA) effect size (LEfSe), Spearman correlation analysis, and phylogenetic investigation of communities by reconstruction of unobserved states (PICRUSt2), were performed on the BMKCloud platform.

### 2.9. Statistical Analysis

All data are presented as mean values ± standard error of the mean (SEM). Data were analyzed by one-way analysis of variance (ANOVA) coupled with Tukey’s post hoc test for multiple comparison or Student t-test using SPSS 23.0 and GraphPad Prism 5. *p* values < 0.05, which are presented with different superscript letters in the figures, were considered statistically significant among groups.

## 3. Results

### 3.1. Tartary Buckwheat Alleviated HFD-Induced Dyslipidemia and Lipid Accumulation

Comparisons of the body weight gain, liver weight, and food intake among the different groups were made (Table 1). After 8 weeks, body weight, liver weight, and the liver weight/body weight ratio were significantly higher for mice on HFD feeding than for those in the ND group. High-dose buckwheat significantly relieved the HFD-induced body weight gain (*p* < 0.05), while the low-dose and medium-dose buckwheat interventions remarkably reduced the elevated levels of liver weight and the liver weight/body weight ratio in the HFD-fed mice (*p* < 0.05). No significant difference in food consumption or energy intake was observed between the HFD group and the tartary buckwheat intervention groups.

**Figure 1:**
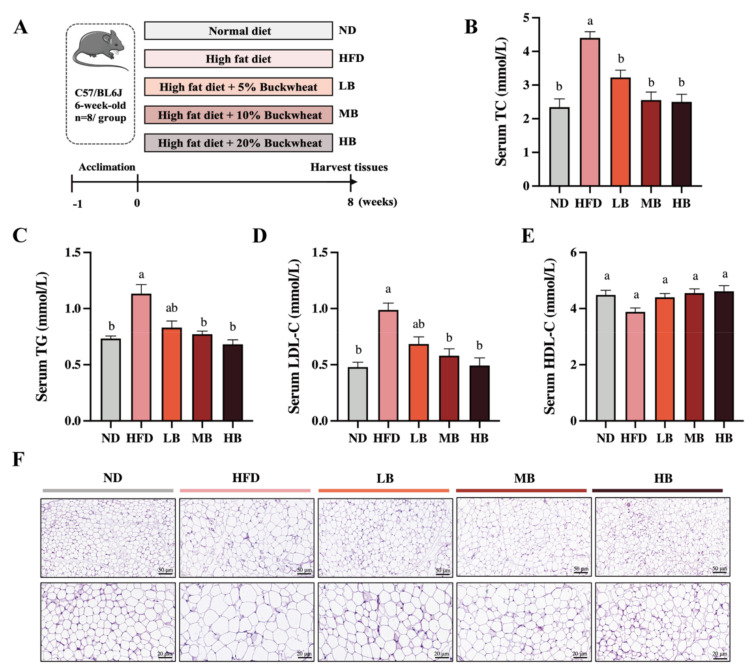
Effects of tartary buckwheat on HFD-induced dyslipidemia and lipid accumulation. (**A**) Flow chart of the animal experiment. (**B**) Serum TC levels. (**C**) Serum TG levels. (**D**) Serum LDL-C levels. (**E**) Serum HDL-C levels. (**F**) H&E staining of epididymis adipose (scale bar, 50 μm and 20 μm). Data are expressed as the mean ± SEM (*n* = 8). The mean value with different letters indicates significant differences (*p* < 0.05).

To determine the functional role of buckwheat in HFD-induced lipid metabolic disorder, C57BL/6 mice were supplemented with LB, MB, and HB under continuous HFD feeding for 8 weeks (Figure 1A). Meanwhile, Figure 1B shows that HFD feeding led to a 1.88-fold increase in serum TC levels compared with the ND group, while the TC levels in the mice fed with LB (3.23 mmol/L), MB (2.56 mmol/L), and HB (2.50 mmol/L) diets were significantly lower than in the HFD-fed mice (4.40 mmol/L; *p* < 0.05 for all analyses). In addition, Figure 1C–E show that HFD feeding notably increased TG and LDL-C levels by 1.55-fold and 2.06-fold, respectively, compared with the control group. TG contents were reduced by 17.00%, 31.86%, and 39.81% in the LB, MB, and HB groups, respectively (*p* > 0.05, *p* < 0.05, and *p* < 0.05), in comparison with the HFD group. LDL-C concentrations were lowered by 30.67%, 41.30%, and 50.00% under the LB, MB, and HB treatments, respectively, compared to the mice fed with HFD (*p* > 0.05, *p* < 0.05, and *p* < 0.05). HDL-C levels were also reduced by 13.50% with HFD feeding compared to the ND group (*p* > 0.05), while the LB, MB, and HB interventions increased HDL-C levels by 13.45%, 17.21%, and 18.93%, respectively, in comparison to the HFD group, but no significant difference was observed. It is worth mentioning that LB, MB, and HB completely reversed the HFD-induced lipid dyslipidemia to levels with no statistical difference compared to the control group, and the preventive effect was dependent on the dosage. Subsequently, the histological results indicate that the epididymal adipocytes in the ND group were regularly arranged, evenly distributed, and uniform in size, whereas the average size of the adipocytes in the mice on HFD feeding was notably enlarged compared with the ND group. Tartary buckwheat attenuated HFD-induced adipocyte hypertrophy, particularly in the HB-fed mice. These findings suggest that the tartary buckwheat intervention alleviated abnormal serum lipid profiles and suppressed adipocyte hypertrophy in response to the high-fat diet.

### 3.2. Tartary Buckwheat Attenuated HFD-Induced Low-Grade Inflammation and Hepatic Steatosis

**Figure 2:**
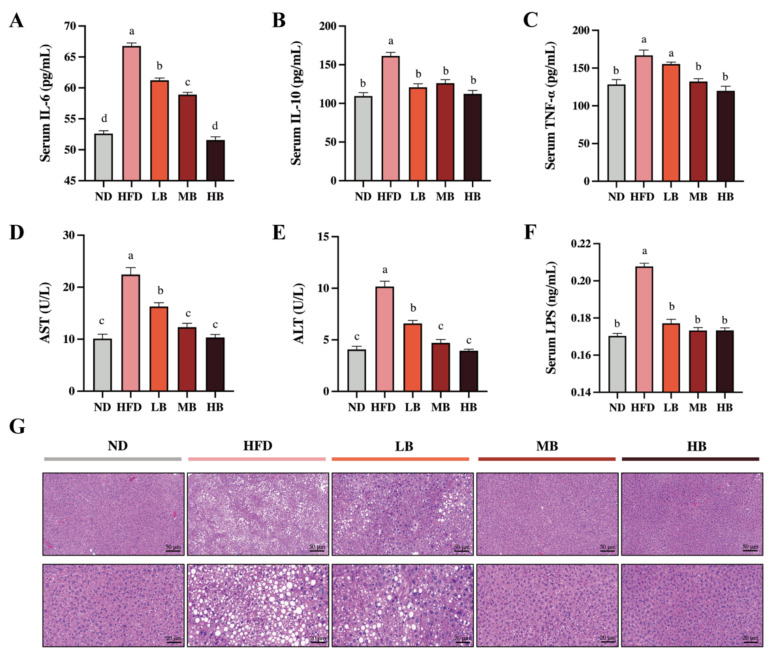
Effects of tartary buckwheat on HFD-induced low-grade inflammation and hepatic steatosis. (**A**) Serum IL-6 levels. (**B**) Serum IL-10 levels. (**C**) Serum TNF-α levels. (**D**) Serum AST levels. (**E**) Serum ALT levels. (**F**) Serum LPS levels. (**G**) Representative images of histological sections of liver tissue (scale bar, 50 μm and 20μm). Data are expressed as the mean ± SEM (*n* = 8). The mean value with different letters indicates significant differences (*p* < 0.05).

Metabolic disorders are usually accompanied by chronic low-grade inflammation [13]. As shown in Figure 2A–C, the levels of IL-6, TNF-α, and IL-10 in the HFD group were 1.27, 1.30, and 1.47 times higher than those in the ND group, respectively. Buckwheat supplementation at low, medium, and high dosages significantly reduced the IL-6 levels in the HFD-fed mice by 8.30%, 11.75%, and 22.79%, respectively (*p* < 0.05 for all analyses). Meanwhile, IL-10 concentrations were decreased by 25.17%, 21.83, and 30.37% under the LB, MB, and HB treatments, respectively, with statistical significance compared to the HFD group. TNF-a levels were also lowered by 6.84%, 20.75%, and 25.16% in the LB, MB, and HB groups (*p* > 0.05, *p* < 0.05, and *p* < 0.05, respectively) compared with the mice on HFD, and no significant difference in TNF-a levels among the MB, HB, and control groups was observed. Additionally, the serum concentrations of ALT and AST showed a 2.50- and 2.22-fold increase with statistical significance in the HFD-fed mice, respectively, compared to the ND group (Figure 2D,E). The LB, MB, and HB treatments dose-dependently reversed the HFD-induced increase in AST and ALT levels (*p* < 0.05 for all analyses). Furthermore, LPS, a major molecular compound found in the outward membrane of Gram-negative germs, has been reported to trigger the release of inflammatory mediators and its related metabolic disturbance. Figure 2F shows that serum LPS contents were significantly increased by HFD consumption (*p* < 0.05), while the serum LPS levels in the LB, MB, and HB groups were remarkably inhibited compared with the HFD group (*p* < 0.05 for all analyses) and recovered to the same level as that found in the ND group. The liver histological evaluation of the control group showed normal liver architecture, integral hepatocytes, and even distribution (Figure 2G), while the livers from the HFD group were characterized by lipid droplets, inflammatory cell infiltration, and blurred cell boundaries. Nevertheless, the livers of the HB-fed mice showed a well-preserved morphology with intact circular nucleated hepatocytes, indicating an enhanced mitigative effect of tartary buckwheat on hepatic steatosis with the dosage of the experimental intervention.

### 3.3. Tartary Buckwheat Modified the Circulating Gut Hormone in HFD-Fed Mice

**Figure 3:**
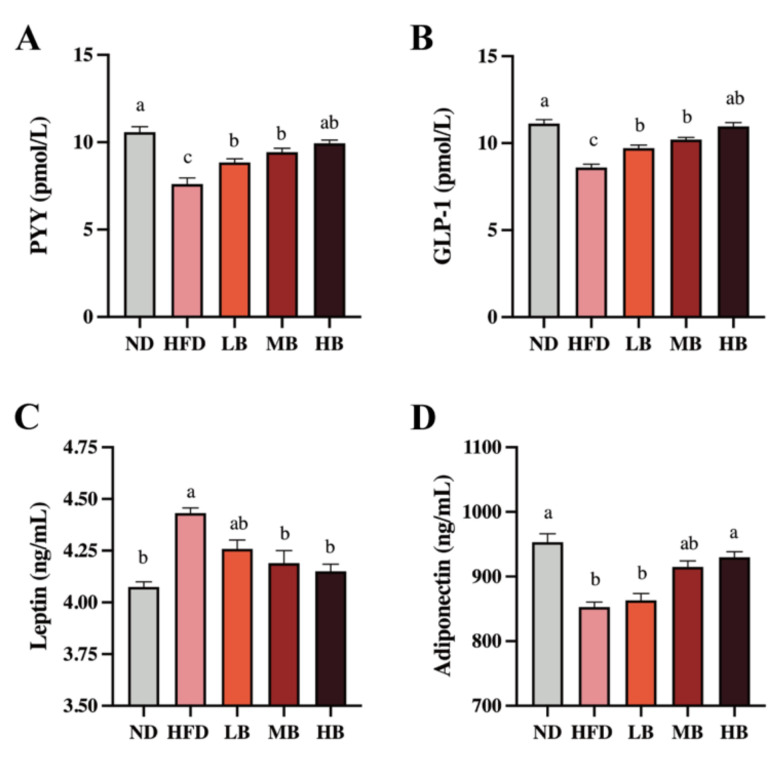
Effects of tartary buckwheat on the circulating gut hormone in HFD-fed mice. (**A**) Serum PYY levels. (**B**) Serum GLP-1 levels. (**C**) Serum leptin levels. (**D**) Serum adiponectin levels. Data are expressed as the mean ± SEM (*n* = 8). The mean value with different letters indicates significant differences (*p* < 0.05).

Gut hormones are crucial to modify food intake, appetite, and energy homeostasis, which regulate lipid deposition, inflammation, and cholesterol metabolism [14]. As shown in Figure 3A, a statistically significant decrease in the PYY level was detected in the HFD group (7.61 pmol/L) compared with that in the ND group (10.58 pmol/L). However, the LB, MB, and HB interventions significantly suppressed the reduction in the PYY levels in the HFD-fed mice (*p* < 0.05 for all analyses). Specifically, the HB intervention restored PYY to control levels. A significant inhibitory effect on GLP-1 secretion was also observed in the mice fed with HFD compared with the ND group (*p* < 0.05), but the LB, MB, and HB treatments elevated the circulating GLP-1 concentrations in a dose-dependent manner, and the data were statistically significant (Figure 3B). Figure 3C also shows that HFD feeding elevated the serum level of leptin by 1.09-fold (*p* < 0.05) compared to the ND group, but the LB, MB, and HB supplementation reversed the HFD-driven over-release of serum leptin by 3.90%, 5.44%, and 6.34% (*p* > 0.05, *p* < 0.05, and *p* < 0.05, respectively). As shown in Figure 3D, adiponectin levels tended to decrease markedly in the HFD-fed mice (852.93 ng/mL) compared to the ND group (953.09 ng/mL; *p* < 0.05). The LB, MB, and HB treatments upregulated adiponectin contents in the HFD-fed mice in response to the dosage, while the variances in the low-/medium-dose intervention groups were not statistically different compared with the HFD-fed mice (*p* > 0.05, *p* > 0.05); a significant difference was noted in the HB group compared to the mice on HFD (*p* < 0.05). These results illustrate that the tartary buckwheat intervention could influence circulating gut hormone levels and that this effect exhibited a dose response to buckwheat consumption.

### 3.4. Tartary Buckwheat Regulated the Expression of Genes and Proteins Linked to Lipid Metabolism

**Figure 4:**
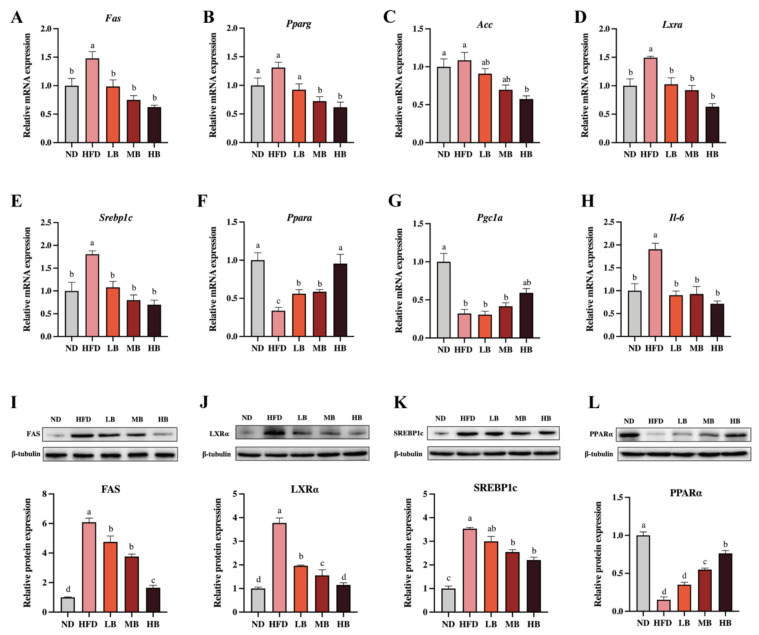
Effects of tartary buckwheat on the expression of genes and proteins linked to lipid metabolism. (**A**–**H**) The relative mRNA expression of hepatic *Fas*, *Pparg*, *Acc*, *Lxra*, *Srebp1c*, *Ppara*, *Pgc1a*, and *Il-6*. (**I**–**L**) Western blot images and the protein expression of hepatic FAS, LXRα, SREBP-1c, and PPARα. Data are expressed as the mean ± SEM (*n* = 8). The mean value with different letters indicates significant differences (*p* < 0.05).

Liver is an important organ involved in lipogenesis and cholesterol metabolism [15]. To deeply probe the underlying mechanism by which the buckwheat regulated lipid metabolism, qRT-PCR and Western blotting were performed to analyze the expression of the lipid-metabolism-related genes and proteins in the liver. As shown in Figure 4A–H, HFD upregulated the mRNA levels of fatty acid synthase (*Fas*), liver X receptor alpha (*Lxra*), sterol regulatory element-binding transcription factor 1 (*Srebp1c*), and *Il-6* by 1.48, 1.49, 1.81, and 1.91 times, respectively, with a statistical difference in data compared to the ND group. Respective 0.34- and 0.32-fold decreases in the mRNA expressions of peroxisome-proliferator-activated receptor alpha (*Ppara*) and peroxisome-proliferator-activated receptor c coactivator 1α (*Pgc1a*) were also observed in comparison with the control group (*p* < 0.05 for all analyses). Meanwhile, the mRNA expressions of peroxisome-proliferator-activated receptor γ1 (*Pparg*) and acetyl-CoA carboxylase (*Acc*) were slightly elevated by HFD feeding, although there was no statistical significance in the data. However, the LB, MB and HB treatments dose-dependently suppressed the mRNA expression of *Pparg* compared with the mice fed HFD (*p* > 0.05, *p* < 0.05, and *p* < 0.05, respectively). The LB, MB, and HB interventions also inhibited the expression of *Acc* and promoted the expression of *Pgc1a* in the HFD-fed mice in a dose-dependent manner, whereas no significant difference was observed in the LB and MB groups. Notably, the tartary buckwheat intervention reversed the differentially expressed genes, including *Fas*, *Lxra*, *Srebp1c*, and *Ppara*, caused by HFD feeding with significant differences (*p* < 0.05 for all analyses). Furthermore, the protein expression levels of FAS, LXRα, SREBP1c, and PPARα also showed a similar trend to the expressions for each corresponding gene.

### 3.5. Tartary Buckwheat Modulated Gut Microbial Profile in HFD-Fed Mice

**Figure 5:**
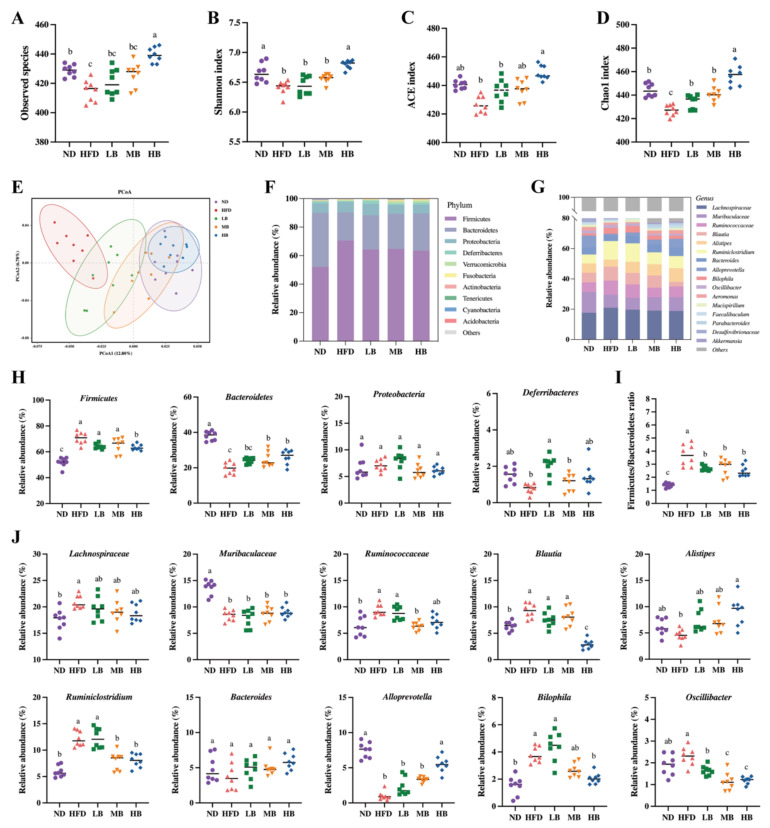
Effects of tartary buckwheat on gut microbial profile. (**A**) Observed species. (**B**) Shannon index. (**C**) ACE index. (**D**) Chao1 index. (**E**) Weighted UniFrac PCoA. (**F**) The gut microbiota composition at the phylum level. (**G**) The gut microbiota composition at the genus level. (**H**) The relative abundance of four representative microbes at the phylum level. (**I**) The ratio of Firmicutes/Bacteroidetes. (**J**) The relative abundance of twelve representative genera. Data are expressed as the mean ± SEM (*n* = 8). The mean value with different letters indicates significant differences (*p* < 0.05).

It is reasonable to speculate that tartary buckwheat may improve lipid metabolism by reprogramming the gut microbiota in mice on HFD feeding because emerging evidence has proven the interaction between diet and gut microbiota [16]. To verify this hypothesis, 16S rRNA gene sequencing was conducted on the intestinal flora of the mice in each group after 8 weeks of the tartary buckwheat interventions. As shown in Figure 5A, we found that the number of observed species in the HFD group (415.00 ± 2.60) was notably lower than that in the ND-fed mice (428.75 ± 1.41) but exhibited an upregulated trend under the LB, MB, and HB treatments (420.63 ± 3.26, 425.75 ± 2.93, and 439.38 ± 1.78, respectively) compared with the HFD group based on operational taxonomic units (OTUs). Notably, the HB intervention increased the observed species in comparison to the HFD group, with a statistically significant difference. Figure 5B,D show that HFD-induced decreases in gut microbiota richness indices (Chao1 and ACE indices) and diversity indices (the Shannon index) were inhibited with the dose-dependent consumption of tartary buckwheat. Specifically, the HB treatment significantly improved the ACE and Shannon indicators (*p* < 0.05 and *p* < 0.05, respectively), while LB, MB, and HB significantly reversed the Chao1 index compared with the HFD group (*p* < 0.05, *p* < 0.05, and *p* < 0.05, respectively). The integral structural differences in the gut microbiota among the five groups were then analyzed using PCoA (Figure 5E). The HFD group mainly clustered in the negative first principal component (PCoA1) and presented an obvious alteration in microbial structure in comparison with the mice on the normal diet. However, the tartary buckwheat supplementation noticeably restored the changes in the microbiota structure induced by HFD, and the extent of recovery was positively correlated with the dosages.

The intestinal flora composition was identified at the phylum and genus levels (Figure 5F,G). The relative abundances of Firmicutes and the Firmicutes/Bacteroidetes ratio were noticeably raised, while Bacteroidetes decreased in the model group (Figure 5H,I). The HB treatment significantly inhibited the abundance of Firmicutes, while MB and HB evidently promoted the abundance of Bacteroidetes compared to the HFD group, and tartary buckwheat reduced the Firmicutes/Bacteroidetes ratio in response to the dosage. Furthermore, Figure 5J indicates that *Lachnospiracea*, *Ruminococcaceae*, *Blautia*, *Ruminiclostridium*, and *Bilophila* were enriched in the HFD group, while the abundances of *Muribaculaceae* and *Alloprevotella* in the model group were significantly lower than those in the ND group. Tartary buckwheat supplementation could partially offset the above-described differential abundance of these strains caused by HFD feeding. Specifically, the HB treatment restrained the enrichment of *Blautia*, *Ruminiclostridium*, *Bilophia*, and *Oscillibacter*, while a modulatory effect of Jinqiao II tartary buckwheat on the growth of *Lachnospiraceae*, *Alistipes*, *Ruminiclostridium*, *Alloprevotella*, and *Oscillibacter* with the buckwheat dosage was also observed. These results suggest that the Jinqiao II tartary buckwheat treatment remarkably reversed the microbial composition in the mice fed with HFD.

**Figure 6:**
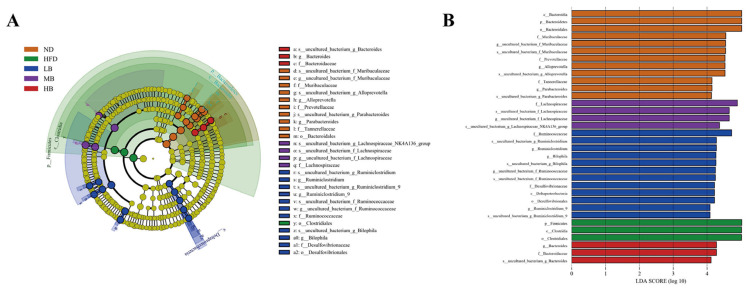
Linear discriminant analysis (LDA) effect size (LEfSe) used to identify the dominant microbial taxa. (**A**) Branch diagram depicting the output of the LEfSe analysis. (**B**) LDA (log10 > 4).

The differential gut microbial taxa among the five experimental groups were derived from the LEfSe analysis. As shown in Figure 6A,B, 34 differentially abundant taxa were detected among the five populations (with LDA scores > 4.0). The bacteria from the genera of *Bacteroides*, *Alloprevotella*, and *Parabacteroides* were dominant organisms in the normal chow-fed mice, while the relative abundance of Firmicutes was enriched in the HFD group. The higher abundances of *Ruminiclostridium* and *Bilophia* were noted in the mice fed with low-dose buckwheat. *Lachnospiraceae* were enriched in the MB group, whereas Bacteroides was the dominant microorganism in the genus category in the HB-fed mice.

### 3.6. Predictive Function Profiling of the Gut Microbiome

**Figure 7:**
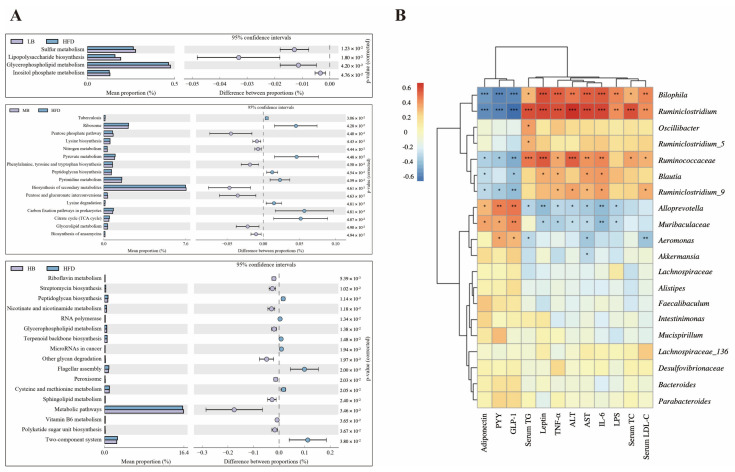
Predictive function profiling of the gut microbiome and correlation analysis. (**A**) PICRUSt analysis based on 16S rRNA sequencing and KEGG database. (**B**) Spearman’s correlation heatmap between gut microbiota and metabolic-disorder-related indices. * *p* < 0.05, ** *p* < 0.01, *** *p* < 0.001.

The PICRUSt analysis was applied to predict the metagenome functions associated with the bacterial communities based on the 16S rRNA sequencing data coupled with the Kyoto Encyclopedia of Genes and Genomes (KEGG) database (Figure 7A). Sulfur metabolism, lipopolysaccharide biosynthesis, glycerophospholipid metabolism, and inositol phosphate metabolism were enriched in the HFD group compared with the mice fed with normal chow. Additionally, when compared to the HFD group, medium-dose buckwheat promoted the pentose phosphate pathway, nitrogen metabolism, the biosynthesis of secondary metabolites, glycerolipid metabolism, and the biosynthesis of ansamycin, but it suppressed pyruvate metabolism, peptidoglycan biosynthesis, pyrimidine metabolism, lysine degradation, and the TCA cycle. Interestingly, high-dose buckwheat upregulated riboflavin metabolism, streptomycin biosynthesis, nicotinate, nicotinamide metabolism, glycerophospholipid metabolism, sphingolipid metabolism, metabolic pathways, and vitamin B6 metabolism, but it downregulated peptidoglycan biosynthesis, terpenoid backbone biosynthesis, cysteine and methionine metabolism, and polyketide sugar unit biosynthesis in comparison with the mice on HFD.

### 3.7. Correlation Analysis between Gut Microbiota and Metabolic Parameters

Correlations between gut microbial composition and metabolic parameters were analyzed using the Spearman correlation test (Figure 7B). *Bilophia* and *Ruminiclostridum* were found to be positively related to the levels of TC, TG, LDL-C, ALT, AST, TNF-α, IL-6, LPS, and leptin but negatively correlated with the circulating concentrations of PYY, GLP-1, and adiponectin. A statistically significant correlation was also observed between the dominant genera, including *Ruminococcaceae* coupled with *Blautia* and lipid metabolism profiling. However, *Alloprevotella* and *Muribaculaceae* were negatively related to AST, ALT, TNF-α, IL-6, LPS, and leptin levels and positively correlated with the contents of PYY, GLP-1, and adiponectin, indicating that these two bacteria possibly play opposite roles to the genera mentioned above, including *Bilophia*, *Ruminiclostridum*, *Ruminococcaceae*, and *Blautia*, in regulating lipid metabolism.

## 4. Discussion

Lipid metabolism disorder has emerged as a severe global public health issue in recent decades [17]. Interestingly, a buckwheat diet has been considered a valid nutritious candidate to prevent lipid metabolism disorders [18]. Common buckwheat has previously been confirmed to protect against nonalcoholic fatty liver disease associated with dyslipidemia; however, a recent UHPLC-MS-based widely targeted metabolomics study reported that higher levels of flavonoids and other functional metabolites contributed to the higher health value of tartary buckwheat than that of common buckwheat [5,19]. Nevertheless, the protective effects, efficient thresholds, and underlying mechanisms of Jinqiao II tartary buckwheat on regulating lipid metabolism and gut microbial dysbiosis remain poorly understood. In our study, lipid metabolism appeared to be perturbed in HFD-fed mice, characterized by dyslipidemia, visceral lipid deposition, and an abnormal serum hormonal profile [20]. Peng et al. reported that the traditional tartary buckwheat intervention significantly reversed liver hepatic steatosis and positively affected the HFD-induced abnormal TG levels [21]. However, the effects of traditional tartary buckwheat on liver weight and the levels of TC, LDL-C, TNF-α, and IL-6 were not statistically significant, indicating the limited prevention of traditional tartary buckwheat. Meanwhile, Jinqiao II tartary buckwheat is pervasively considered to exhibit an optimized health value with higher levels of bioactive components and amino acids than the cultivars. Consistently, we found that Jinqiao II tartary buckwheat at different dosages (5%, 10%, and 20%) lowered the increase in the HFD-induced serum lipid biochemical profiles, including serum TC, TG, and LDL-C levels; attenuated the abnormal liver function represented by AST and ALT levels; partially alleviated hepatic steatosis and fat accumulation; and suppressed the over-release of inflammatory cytokines, including TNF-α and IL-6, with the dosages of the intervention. Based on the previously mentioned analyses, dose-sensitive changes in the related physiological indices were observed, and the HB treatment exerted the optimal outcomes, which is consistent with our previous speculations. HFD, which can result in an increased intestinal permeability and leaky gut, has been considered to promote LPS release and, thus, induce low-grade systemic inflammation. The 8-week tartary buckwheat treatment was shown to reduce plasma LPS concentration and systemic inflammation in our study. These results suggest that the tartary buckwheat at low, medium, and high doses ameliorated the lipid metabolism in the HFD-induced obese mice.

Recently, growing evidence has demonstrated that intestinal hormones are key regulators in energy balance and glucose homeostasis, which are closely associated with metabolic disturbance [22]. Compared with HFD-fed mice, a decreased food intake was calculated in the MB and HB groups, suggesting a potential role of tartary buckwheat in appetite control, but this was not statistically significant in our study. To probe the mechanism behind the protective effect of Jinqiao II tartary buckwheat at different dosages on HFD-caused lipid metabolic disruption, the circulating hormonal profile, including PYY, GLP-1, leptin, and adiponectin levels, was then determined. These gut hormones have been proven to stimulate the appetite-modulating circuits in the brain and trigger satiety in the hypothalamus [23]. Specifically, leptin, a hormone released by adipocytes, can regulate energy expenditure and stimulate macrophages to secrete inflammatory factors [24]. Excessive levels of circulating leptin are generally related to an increase in subcutaneous fat and obesity [25]. Additionally, adiponectin, an adipocytokine secreted from adipocytes, is known to contribute to maintaining energy homeostasis and glucolipid metabolism [26]. PYY and GLP-1, secreted by enteroendocrine cells, have also been documented to modulate appetite and inhibit food intake [27]. Our results imply that tartary buckwheat can inhibit leptin release and elevated circulating levels of PYY, GLP-1, and adiponectin, which were critically dependent on the intervention dosage. The resulting satiety signals transmitted to the central nervous system via the vagus nerve were integrated and then influenced feeding behavior, intestinal transport, and the interaction between food matrices and gut enzymes.

Liver is a primary organ with multiple vital functions, including glucose homeostasis and lipid metabolism [28]. A high-fat diet has been presumed to accumulate lipid droplets and intensify structural and functional changes in the liver, ultimately leading to abnormal hepatic metabolism [29]. Accordingly, this research quantified the pivotal genes related to lipid metabolism and cholesterol synthesis in the liver using mRNA (qRT-PCR) and protein (western blot) expression analyses. *Srebp1c*, a key transcription factor, contributes to triglyceride biosynthesis by altering the gene expressions of *Acc* and *Fas*, while *Lxra* has been found to mediate lipogenesis by promoting *Srebp1c* expression [30]. Additionally, it is well established that *Ppara* may affect the relevant genes in lipid metabolism, enhance lipid oxidation, and inhibit excessive lipid accumulation in the liver [31]. *Pparg*, however, can facilitate adipocyte differentiation, promote fat biosynthesis, and attenuate inflammation [32]. *Pgc1a*, a required regulator in brown adipocytes, has been suggested to be involved in adipose tissue thermogenesis and mitochondrial biogenesis [33]. In this study, the MB and HB treatments observably restrained the expression level of *Pparg*, whereas the HB supplementation notably downregulated the mRNA expression of *Acc* and upregulated the gene expression of *Pgc1a*. Furthermore, the mRNA expressions of *Fas*, *Lxrα*, *Srebp1c*, and *Ppara* were found to be reversed in response to the tartary buckwheat consumption. The trends in the protein levels of FAS, LXR, SREBP1c, and PPARα were similar to those found in the mRNA expression, which is in accordance with a prior finding about the prevention of a probiotic-fermented rice buckwheat on HFD-induced hyperlipidemia in mice [34].

Whole grains have previously been proven to modulate intestinal flora composition [35]. The relationship between the beneficial effects of tartary buckwheat at different doses and the gut microbial profile was further explored in this study using 16S rRNA sequencing. We noticed that HFD feeding repressed both the richness indices (ACE index, Chao1 index, and observed species) and diversity indicator (the Shannon index), but this was offset by tartary buckwheat in response to the dosages. Our finding also shows that the characteristics considered indicative of obesity and metabolic syndrome, including the higher abundance of Firmicutes, the suppressed abundance of Bacteroidetes, and the elevated Firmicutes/Bacteroidetes ratio, were observed in the HFD group [36]. However, the high-dosage tartary buckwheat intervention restored the HFD-induced Firmicutes enrichment and Bacteroidetes expression reduction, while tartary buckwheat showed an inhibitory effect on the Firmicutes/Bacteroidetes ratio at low, medium, and high consumption. Tartary buckwheat was also proved to alter the intestinal microbiota composition and further reshape the gut microbiota in the HFD group.

In our research, *Lachnospiraceae*, *Ruminococcaceae*, *Blautia*, *Ruminiclostridium*, and *Bilophila* were enriched, while *Muribaculaceae* and *Alloprevotella* were suppressed in the HFD group in comparison to the mice on the normal control diet. However, the tartary buckwheat partially reversed the differential abundance changes in these strains caused by HFD feeding. It is worth noting that a positive dose-dependent manner in the intervention effect of the tartary buckwheat on the relative abundance of *Lachnospiraceae*, *Alistipes*, *Ruminiclostridium*, *Alloprevotella*, and *Oscillibacter* was also observed. Theoretically, the gut microbiome is a critical component of the intestinal immune barrier and is highly associated with maintaining intestinal balance. Intestinal microbiome dysbiosis may disrupt the gut epithelial barrier and increase intestinal permeability. This leaky gut may permit the translocation of bacteria and their metabolites into the systemic circulation, leading to systemic microinflammation and metabolic endotoxemia [37]. Our findings align with previous observations that HFD increased circulating LPS levels and inflammatory cytokine release. We also verified the positive correlation between lipid metabolism parameters and the abundances of *Bilophila*, *Ruminiclostridium*, and *Oscillibacter*, which can exacerbate HFD-induced metabolic dysfunction and promote the development of intestinal inflammation as a common LPS-producing bacteria [38,39,40]. Conversely, *Muribaculaceae*, known to reduce serum LPS levels and maintain intestinal epithelial health, was negatively correlated with metabolic indicators based on our results [41]. We noticed that the HB intervention evidently reduced the relative abundance of *Bilophila* and prevented HFD-induced lipid metabolism disturbances, which is consistent with previous findings [42]. *Lachnospiraceae*, a bacterium associated with digestive tract function, has been found to contribute to fat accumulation and an abnormal lipid profile [43]. Moreover, we noted a significant enrichment of *Lachnospiraceae* in the HFD group. Although the differences in the dose response were not statistically significant, the tartary buckwheat intervention exerted a preventive effect in a positive dose-dependent manner. *Alloprevotella* was promoted by the tartary buckwheat intervention in a dose-sensitive manner, with proven preventive effects on obesity-related indices [44]. Previously, *Alistipes* has been documented to release anti-inflammatory metabolites and maintain gut permeability by regulating the tight junction protein [45]. Consistently, *Alistipes* had a low abundance in the LB and MB groups but a high abundance with the HB treatment. HFD feeding can also enrich *Ruminococcaceae*, which are positively correlated with the released pro-inflammatory factors; then, the raised intestinal permeability promotes the translocation of microorganisms to the circulation, resulting in systemic inflammation, which is in line with the results from our correlation analysis [46]. To summarize, the tartary-buckwheat-mediated ameliorative effects on the metabolic situation, which occurred in a dose-sensitive manner, may be related to the enrichment of beneficial gut bacteria and the decreased abundance of potentially pathogenic bacterial taxa in the gut microbial community.

In our study, 8-week Jinqiao II tartary buckwheat was proved to neutralize the adverse negative effects caused by a high-fat diet. Specifically, high-dose tartary buckwheat exerted optimal outcomes. The preventive effect on lipid metabolism disorders and gut microbiota dysbiosis in response to the dosages of a newly cultivated variety of tartary buckwheat was first revealed. Our findings might offer a new perspective for the prevention of HFD-induced lipid metabolism disorder and related metabolic diseases. Nevertheless, mechanical studies are needed to identify the causality in the interaction between gut microbiota and tartary-buckwheat-mediated regulation on lipid metabolism disorder. Further clinical validation is needed to verify the long-term efficacy and to draw a supportive nutritional recommendation.

## 5. Conclusions

Overall, this study suggests that the 8-week Jinqiao II tartary buckwheat intervention alleviated HFD-induced lipid metabolism disorders, including dyslipidemia, lipid deposition, low-grade inflammation, hepatic steatosis, abnormal circulating hormone release, and differentially expressed genes and proteins related to lipid metabolism. These preventive effects were dose-dependent, and high-dose tartary buckwheat exerted optimal outcomes. The Jinqiao II tartary buckwheat treatment also improved the gut microbial richness and diversity; reshaped the intestinal flora composition; promoted beneficial bacteria, such as *Alistipes* and *Alloprevotella*; and inhibited the growth of pathogenic bacterial taxa, including *Ruminococcaceae*, *Blautia*, *Ruminiclostridium*, *Bilophila*, and *Oscillibacter*. Collectively, this discovery may suggest a promising future development of functional food choices from novel Jinqiao II tartary buckwheat to prevent lipid metabolism disorder.

## Figures and Tables

**Table 1:** Body weight, liver tissue index, and food intake of the mice.

Mouse Group	Body		Liver		Food Intake	
	Final Weight (g)	Weight Gain (g)	Weight (g)	Ratio (%)	Consumption (g/d)	Energy Intake (kcal/d)
ND	23.62 ± 0.68 ^b 1^	6.20 ± 1.45 ^b^	0.72 ± 0.02 ^b^	2.64 ± 0.10 ^b^	2.61 ± 0.16 ^a^	10.05 ± 0.62 ^b^
HFD	31.32 ± 1.11 ^a^	14.24 ± 0.84 ^a^	1.40 ± 0.02 ^a^	4.48 ± 0.07 ^a^	2.43 ± 0.19 ^a^	12.73 ± 0.98 ^a^
LB	27.02 ± 0.23 ^a^	10.20 ± 0.33 ^ab^	0.93 ± 0.04 ^b^	3.38 ± 0.19 ^b^	2.44 ± 0.06 ^a^	12.79 ± 0.29 ^a^
MB	24.98 ± 0.69 ^ab^	7.98 ± 1.39 ^ab^	0.86 ± 0.02 ^b^	3.47 ± 0.09 ^b^	2.38 ± 0.10 ^a^	12.47 ± 0.51 ^a^
HB	23.05 ± 0.49 ^b^	6.45 ± 1.01 ^b^	0.73 ± 0.03 ^b^	3.19 ± 0.14 ^b^	2.24 ± 0.16 ^a^	11.74 ± 0.82 ^a^

^1^ The mean value with different letters indicates significant differences (*p* < 0.05).

## Data Availability

Data is contained within the article or Supplementary Material.

## Supplementary Materials

The following supporting information can be downloaded at: https://www.mdpi.com/article/10.3390/foods11193028/s1, Table S1: Detailed information of the mouse diet; Table S2: Primer sequences for qRT-PCR.
