# Supplementary material for: Tartary Buckwheat (Fagopyrum tataricum) Ameliorates Lipid Metabolism Disorders and Gut Microbiota Dysbiosis in High-Fat Diet-Fed Mice
Source: Foods. 2022 Sep 29;11(19):3028. doi: 10.3390/foods11193028 (PMC9563051; doi:10.3390/foods11193028)
Supplement: Supplementary file 1 [file foods-11-03028-s001.zip › foods-1863305-supplementary.pdf]

Table S1. Detailed information of the mouse diet

**Product**	**XTCON50J**		**XTHF60**		**XTHF60（5% buckwheat）**		**XTHF60（10% buckwheat）**		**XTHF60（20% buckwheat）**	
**Nutrients**	**gm%**	**Kcal%**	**gm%**	**Kcal%**	**gm%**	**Kcal%**	**gm%**	**Kcal%**	**gm%**	**Kcal%**
Protein	19.2	20.0	26.0	20.0	26.0	20.0	26.0	20.0	26.0	20.0
Carbohydrate	67.3	70.0	26.0	20.0	26.0	20.0	26.0	20.0	26.0	20.0
Fat	4	10.0	35	60.0	35	60.0	35	60.0	35	60.0
Total		100.0		100.0		100.0		100.0		100.0
Kcal/gm	3.85		5.24		5.24		5.24		5.24	
**Ingredient**	**gm**	**Kcal**	**gm**	**Kcal**	**gm**	**Kcal**	**gm**	**Kcal**	**gm**	**Kcal**
Casein	200.0	800.0	200.0	800.0	195.4	781.4	190.7	762.9	181.4	725.8
L-Cystine	3.0	12.0	3.0	12.0	3.0	12.0	3.0	12.0	3.0	12.0
Corn Starch	506.200	2025	0.000	0	0.000	0	0.000	0	0.000	0
Maltodextrin 10	125	500	125	500	95	380	65	259	5	18
Sucrose	68.800	275	68.800	275	68.800	275	68.800	275	68.800	275
Buckwheat Fat (2%)					0.774	7	1.548	14	3.095	28
Buckwheat Protein (12%)					4.640	19	9.280	37	18.560	74
Buckwheat Starch (70%)					27.000	108	54.000	216	108.000	432
Buckwheat Fiber (8%)					3.100	0	6.200	0	12.400	0
Other Buckwheat Substances (8%)					3.100	0	6.200	0	12.400	0
Cellulose	50	0	50	0	47	0	44	0	38	0
Soybean Oil	25	225	25	225	24	218	23	211	22	197
Lard	20	180	245	2205	245	2205	245	2205	245	2205
Mineral Mix S10026	10.0		10.0		10.0		10.0		10.0	
DiCalcium Phosphate	13.0		13.0		13.0		13.0		13.0	
Calcium Carbonate	5.5		5.5		5.5		5.5		5.5	
Potassium Citrate，1 H2O	16.5		16.5		16.5		16.5		16.5	
Vitamin Mix V10001	10.0	40	10.0	40	10.0	40	10.0	40	10.0	40
Choline Bitartrate	2.0		2.0		2.0		2.0		2.0	
**Total**	**1055**	**4057**	**773.80**	**4057**	**773.80**	**4045**	**773.80**	**4032**	**773.80**	**4008**

Table S2. Primer sequences for qRT-PCR

Gene	Forward primer（5’→3’）	Reverse primer（5’→3’）
Pparg	CCAGCATTTCTGCTCCACAC	ATTCTTGGAGCTTCAGGCCA
Pgc1a	AGCCGTGACCACTGACAACGAG	GCTGCATGGTTCTGAGTGCTAAG
Acc	GGCAGCAGTTACACCACATAC	TCATTACCTCAATCTCAGCATAGC
Il-6	ACTTCCATCCAGTTGCCTTCTTG	TGTTGGGAGTGGTATCCTCTGTG
Fas	GCTGCGGAAACTTCAGGAAAT	AGAGACGTGTCACTCCTGGACTT
Ppara	TGCAGCCTCAGCCAAGTTGAA	TCCCGAACTTGACCAGCCA
Srebp1c	CTGGTGAGTGGAGGGACCAT	GACCGGTAGCGCTTCTCAAT
Lxra	TCAGAAGAACAGATCCGCTTG	CGCCTGTTACACTGTTGCT
β-actin	ACAGCAGTTGGTTGGAGCAA	ACGCGACCATCCTCCTCTTA

Pparg, peroxisome proliferator-activated receptor γ1; *Pgc1a*, peroxisome proliferator-activated receptor c coactivator 1α; *Acc,* acetyl-CoA carboxylase; *Il-6*, interleukin-6; *Fas*, fatty acid synthase; *Ppara*, peroxisome proliferator-activated receptor alpha; *Srebp1c*, sterol regulatory element-binding protein-1c; *Lxra*, liver X receptors α;
